# Effects of different temperatures on growth and intestinal microbial composition of juvenile *Eriocheir sinensis*

**DOI:** 10.3389/fphys.2023.1163055

**Published:** 2023-07-13

**Authors:** Meng Liang, Wenrong Feng, Xue Chen, Yongkai Tang, Jianlin Li, Wenjing Li

**Affiliations:** ^1^ Wuxi Fisheries College, Nanjing Agricultural University, Wuxi, China; ^2^ Key Laboratory of Freshwater Fisheries and Germplasm Resources Utilization, Ministry of Agriculture and Rural Affairs, Freshwater Fisheries Research Center, Chinese Academy of Fishery Sciences, Wuxi, China; ^3^ National Demonstration Center for Experimental Fisheries Science Education, Shanghai Ocean University, Shanghai, China; ^4^ Jiangsu Haorun Biological Industry Group Co., Ltd, Taizhou, China

**Keywords:** temperature, *E. sinensis*, survival, growth performance, intestinal bacterial composition

## Abstract

The change in temperature will change the composition of intestinal microorganisms of juvenile *Eriocheir sinensis*, and the composition of intestinal microorganisms will affect the growth and development of juvenile crabs. In order to explore the relationship between intestinal microorganisms and growth of *E. sinensis* at different temperatures, the status of growth and intestinal microflora of juvenile *E. sinensis* reared at different water temperatures (15 °C, 23 °C, and 30 °C) were compared in this study. The results showed that the respective survival rate of juvenile *E. sinensis* in the three water temperature groups was 100%, 87.5%, and 64.44%. Moreover, the molting rate increased with an increase in water temperature, which was at 0%, 10%, and 71.11% for the three respective temperature groups. The average weight gain rate showed an overall increasing trend with the increase of water temperature. Moreover, the final fatness of the crabs in the 30 °C water temperature group was significantly lower than that in the 15 °C and 23 °C groups (*p* < 0.05); there was no significant difference in the liver-to-body ratio among the three groups. The results of the alpha diversity analysis of the 16S rRNA data revealed that there was no significant difference in the intestinal microbial abundance among the three water temperature groups; however, the intestinal microbial diversity in the 23 °C water temperature group was significantly lower than that in the 15 °C and 30 °C groups. At the phylum level, the dominant flora of the three groups was Firmicutes, Proteobacteria, and Bacteroidota. At the genus level, the abundance of *Parabacteroides* and *Aeromonas* in the intestine of the crabs in the 30 °C water temperature group was significantly higher than that in the 15 °C and 23 °C groups (*p* < 0.05). The function prediction showed that the main functional diversity of intestinal microflora of juvenile *E. sinensis* in the three water temperature groups was similar and mainly involved in metabolic-related functions, but there were still differences in the effects of water temperature on functional pathways such as metabolism, immunity, and growth among each group, either promoting or inhibiting. In conclusion, different water temperatures can affect the composition and function of intestinal flora of *E. sinensis*, and 23 °C–30 °C is the optimal water temperature for the growth of juvenile *E. sinensis*.

## 1 Introduction

Crustaceans are variable temperature animals with weak thermoregulation ability and a body temperature that directly depends on the surrounding ambient temperature. Therefore, water temperature is one of the most important environmental factors in the survival of aquatic crustaceans as it directly affects metabolism, growth, and molting (Gong et al., 2015; Wittmann et al., 2018; Shields, 2019). A study showed that a change in temperature directly affected the routine metabolic rate of *Litopenaeus vannamei*, and the rate increased significantly with the increase in temperature, both for fed and starved shrimp species (Walker et al., 2011). Another study demonstrated that temperature affected the growth, feeding rate, and feed conversion rate (FCR) of juvenile *L. vannamei.* The growth and feeding rate of *L. vannamei* increased directly with the increase in temperature. In addition, the data showed that the optimal temperature (the fastest growing temperature) was specific to size and decreased with the increase of the shrimp size (Wyban et al., 1995). In a certain temperature range, the development and growth rate of shrimp and crab larvae accelerate with the increase in temperature. With the increase in temperature, the larger the body length and weight of *L. vannamei*, the higher is its growth rate (Al‐Masqari et al., 2022). The growth rate of *Macrobrachium nipponense* in a temperature ranged 22 °C–32 °C was significantly higher than that at 16 °C and 20 °C (Wang et al., 2006). The embryonic development process of *Procambarus clarkii* accelerated, and the larval development time shortened with the increase in temperature (Jin et al., 2019). This phenomenon has also been reported in the studies of *Panulirus ornatus* (Sachlikidis et al., 2010), *Eogammarus sinensis* (Xue et al., 2021), and *Penaeus japonicu*s (Coman et al., 2002).

Neuparth et al. found that temperature was the most significant factor affecting the life cycle of *Gammarus locusta*. Under ambient temperature, high temperature shortened the generation time, increased the individual growth rate, accelerated sexual maturity, and increased the population density of *G. locusta* (Neuparth et al., 2002). Moreover, there are many reports on the effect of temperature in the growth and behavior of *E. sinensis*. When the water temperature is lower than 5 °C, *E. sinensis* enters a dormant state; at 6 °C–19 °C, it will crawl with low frequency and eat at a small amount. When the water temperature is 20 °C–26 °C, the food intake is the most exuberant (Dong et al., 2010). The results showed that the growth rate of juvenile *E. sinensis* (0.06–0.15 g) at 28 °C and 30 °C was significantly faster than that at 18 °C and 22 °C. The first molting period was significantly shortened with the increase in temperature. The optimum water temperature for juvenile growth and molting of *E. sinensis* is 28 °C–30 °C (Yuan et al., 2017). First-year juvenile *E. sinensis* have weak tolerance to high temperature. When the water temperature exceeds 30 °C, they will gradually die in a short period of time (Peng et al., 2019).

Intestinal microorganisms refer to the large number of microorganisms in the intestines of humans and animals. These microorganisms promote the growth and development of the host to help in food digestion, detoxify harmful molecules, provide essential nutrients, combat invasive pathogens, and regulate immunity (Engel and Moran, 2013). The development of the intestinal tract is also strongly affected by the existence and composition of intestinal microflora (Sommer and Bäckhed, 2013; Robertson et al., 2019). Genetic factors, lifestyle, and natural environment such as diet and ambient temperature (Gould et al., 2018; Osadchiy et al., 2019) affect the composition of microorganisms (Zmora et al., 2019). Changes in temperature can lead to changes in the function of intestinal flora, thus affecting the digestion and absorption of food (Sun et al., 2022). In a certain temperature range, the increase in temperature helps in colonizing some beneficial bacteria in the intestinal tract and promotes the digestion and absorption of the host. However, when the temperature is very high, it will lead to an increase in the content of pathogenic bacteria in the intestinal tract and damage to the intestinal tract, thus affecting the growth and development of the host and even leading to the death of the host. Some studies have shown that a moderate increase in water temperature promoted the proliferation of beneficial bacteria such as *Bifidobacterium* and *Lactobacillus* in the gut of *L. vannamei* to inhibit the colonization of pathogens, improving the digestion and absorption of food by the host (Yin et al., 2004). However, the rise in *Vibrio*, *Candidatus Bacilloplasma*, and *Photobacterium* spurred the invasion of pathogenic bacteria when the water temperature reached 30 °C, which hampered normal growth and development of the shrimp and ultimately resulted in death (Al‐Masqari et al., 2022). Due to the diversity of living environments, feeding habits, and intestinal structure of aquatic animals, the dominant groups of microorganisms in their intestines vary. Firmicutes, Actinomycetes, Fusobacteria, and Proteobacteria dominate the intestinal tract of shrimp, while the proportion of *Bacteroides* is relatively low (Rungrassamee et al., 2014; Zeng et al., 2017). Compared with higher animals, fewer counts of Bacteroidota are observed in the intestinal tract of aquatic animals, while Fusobacteria and Proteobacteria predominate (Kostic et al., 2013).

There are also some reports on the relationship between temperature, gut microbiota, and host growth and development. Temperature variation structures the composition and function of gut microbiomes in animals (Sepulveda and Moeller, 2020). The destruction of gut microbiota by heat stress may ultimately affect the growth and reproductive characteristics of the host (Dubos and Schaedler, 1960; Gould et al., 2018; Moeller et al., 2019). Changes in ambient temperature could more easily alter the diversity and composition of gut microbiota of ectotherms, potentially affecting their development and health (Fontaine et al., 2018; Huyben et al., 2018; McMunn et al., 2022). Research has found that gut microbial diversity is reduced in salamanders and lizards when they experienced increases in environmental temperatures, which reduced digestive performance in the former and decreased survival in the latter (Bestion et al., 2017; Fontaine et al., 2018; Zhu et al., 2021).

There have been several reports on intestinal microorganisms of *E. sinensis*; however, these were mainly based on the classification and identification of dominant intestinal microflora. *E. sinensis* has high phenotypic plasticity, and the composition of intestinal microbial community is significantly affected by the habitat environment (Su et al., 2020; Chen et al., 2021a). Chen et al. (2015) studied the microflora in the intestines of *E. sinensis* cultured in Tai Lake and found that Proteobacteria, Bacteroidetes, and Tenericutes were the dominant groups. Guan et al. (2021) found that the dominant flora in the intestinal microflora of *E. sinensis* in an alkaline region in Northwest China was Firmicutes. In addition, there is relatively little research on the effect of temperature on the relationship between gut microbiota and growth of *E. sinensis*. Studies have shown that under acute heat stress, the immune function and intestinal microbial community of *E. sinensis* were affected. At 32 °C, the abundance of the beneficial bacteria *Candidatus*, *Hepatoplasma*, and *Marinifilum* decreased significantly, while the abundance of the pathogenic bacteria *Rhodococcus* and *Morganella* increased significantly, thereby affecting the normal growth and development of *E. sinensis* (Li et al., 2023).

With the global warming and the increase of various extreme weather events in recent years, there are more and more studies on the effects of temperature on crustaceans. However, only few studies explored the environmental factors (e.g., temperature and salinity) that may affect the intestinal microorganisms of juvenile *E. sinensis*. The growth and development of juvenile *E. sinensis* is closely related to temperature, and the change in temperature will lead to changes in the composition of intestinal flora of juvenile *E. sinensis*, which has a great impact on the growth and survival of the host. Currently, no study has fully studied whether the growth difference is caused by the change in intestinal microflora due to the change in temperature. In this study, three different temperature groups (15 °C, 23 °C, and 30 °C) were used to compare and analyze the growth and abundance of intestinal microflora of juvenile *E. sinensis*. The purpose of this study was to explore the effect of temperature on intestinal microorganisms, and evaluate the relationship between intestinal flora and growth. The results of this study can provide a reference basis for optimizing the breeding conditions of juvenile *E. sinensis*, promoting the healthy and rapid growth of juvenile crabs, and preventing and diagnosing crab diseases.

## 2 Materials and methods

### 2.1 Feeding and management of experimental crabs

Juvenile E. sinensis (10.4976 ± 0.38 g) were obtained from Yangcheng Lake Shrimp and the Crab Green Culture Base of Freshwater Fisheries Research Center of Chinese Academy of Fisheries Sciences. All crabs were allowed to adapt to laboratory conditions for 1 week inside a transparent glass tank (100 cm × 45 cm × 50 cm). During this period, the water quality parameters were maintained at water depth 15 cm, temperature 15°C ± 1 °C, pH 8.0 ± 0.5, dissolved oxygen ≥5.0 mg/L, ammonia ≤0.1 mg/L, and nitrite ≤0.05 mg/L (Pan et al., 2020). Juvenile *E. sinensis* was fed at 14:00 daily, and the remaining feed and feces were retrieved at 8:00 the next day. One-third of the water in the tank was changed regularly to keep the water quality stable.

### 2.2 Experimental design and sample collection

Three temperature gradients of 15 °C (low), 23 °C (mid), and 30 °C (high) were set. The temperature was adjusted using the temperature control electric heating rod and monitored in real time using a thermometer. A total of 120 juvenile *E. sinensis* were randomly divided into three groups: the low-temperature group (*n* = 35), middle-temperature group (*n* = 40), and high-temperature group (*n* = 45). Each temperature group of juvenile crabs was then randomly divided into three glass tanks for experiments. The crabs were individually fed based on 2% of the total mass at a normal time daily for 30 days, and the residual bait and feces were cleaned up every day.

#### 2.2.1 Growth index determination

Before the start of the feeding experiment, the crabs fasted for 24 h, and their body mass, body length, body width, and body height were measured. Ten crabs were randomly sampled; their hepatopancreas and body mass were measured to calculate the initial liver-body ratio. After fasting for 24 h at the end of the feeding experiment, the body mass, body length, body width, and body height were measured, and the weight gain rate was calculated. Ten crabs were randomly selected from each group, and the hepatopancreas and body mass were measured to calculate the final liver-body ratio. During the culture experiment, the number of dead crabs was recorded to calculate the survival rate. The molting date and times for each experimental crab were recorded, and the growth index was counted.

#### 2.2.2 Sample collection

The crabs fasted for 24 h before sampling. Twelve crabs were selected for each water temperature group, and the intestines were collected and mixed into six replicates for sequencing and analysis of intestinal microbial communities. In a sterile environment, the intestines were removed with tweezers and placed in a 1.5-mL centrifuge tube. The samples were flash frozen with liquid nitrogen and stored in a refrigerator at −80 °C. The animals used in the experiment were approved by the Freshwater Fisheries Research Center, and all experimental procedures were performed in accordance with the Animal Protection Guide.

### 2.3 Extraction and PCR amplification of total DNA of intestinal flora

The genomic DNA of the sample was extracted using the CTAB method. The purity and concentration of the extracted DNA were detected by agarose gel electrophoresis. Subsequently, sufficient amount of DNA was taken in the centrifuge tube and diluted to 1 ng/μL with sterile water. Using the diluted genomic DNA as the template, the specific primers with barcode, Phusion ®High-Fidelity PCR Master Mix with GC Buffer of New England Biolabs, and high efficiency and high-fidelity enzyme were selected according to the amplification region to ensure the amplification efficiency and accuracy. The PCR products were detected by 2% agarose gel electrophoresis, and the target bands were recovered using the gel recovery kit (QIAGEN). The library was constructed using the NEBNext^®^ Ultra IIDNA Library Prep Kit, and the constructed library was quantified using Qubit and Q-PCR. The library was sequenced using the NovaSeq 6000 System.

### 2.4 Sequencing data processing and analysis of intestinal microorganisms

According to the barcode sequence and PCR amplification sequence, the sample data were separated from the offline data, and the barcode and primer sequences were truncated. The sample reads were then spliced using FLASH (V1.2.11, http://ccb.jhu.edu/software/FLASH/) (Magoč and Salzberg, 2011) to obtain the raw tags. Quality control of the raw tags was assessed using fastp, and high-quality clean tags were obtained. The USEARCH software application is used to compare the clean tags with the database to detect and remove chimerism (Haas et al., 2011) to obtain the final effective tags. The DADA2 module of QIIME2 software was used to reduce noise and filter out the sequences with abundance <5, obtaining the final amplified subsequence variation (ASV) and feature table. The classify-sklearn module of QIIME2 software was used to compare the ASVs with the database to obtain the corresponding species information. Observed_otus, Shannon, Simpson, chao1, goods_coverage, dominance, and pielou_e indices were calculated using QIIME2 software. Moreover, QIIME2 software was used to calculate the UniFrac distance, and R software was used to perform PCA, PCoA, and NMDS dimensionality reduction maps. Subsequently, adonis and anosim functions in QIIME2 software were used to analyze the significant differences in community structure between groups. LEfSe or R software (R Foundation for Statistical Computing, Vienna, Austria) was used to analyze the significant species differences between groups. SPSS version 23.0 (IBM Corp., Armonk, NY, United States) was used to analyze the statistical differences.

## 3 Results

### 3.1 Growth performance of juvenile *E. sinensis* at different water temperatures

The survival rate of the experimental crabs in the 30-day feeding experiment gradually decreases with the increase in water temperature. Specifically, the survival rate of experimental crabs at 15 °C water temperature was 100% and dropped to 64.44% at 30 °C. The molting rate was proportional to the water temperature. The experimental crab in the 15 °C water temperature group did not molt; however, 10% and 71.11% of the crabs molted in the 23 °C and 30 °C water temperature groups, respectively. With the increase in water temperature, the average weight gain rate showed an overall increasing trend and the differences between groups were significant. The final fatness of crabs in the 30 °C water temperature group was significantly lower than that in the 15 °C and 23 °C (*p* < 0.05) water temperature groups, and there was no significant difference in the liver-body ratio among the three groups (*p* < 0.05). However, compared with the initial liver-body ratio, the final liver-body ratio showed a downward trend. The molting and growth characteristics of juvenile *E. sinensis* are shown in (Table 1).

**TABLE 1:** Molting and growth characteristics of juvenile *E. sinensis* cultured under different water temperatures.

Water temperature/°C	15 °C	23 °C	30 °C
Stocking number	35	40	45
Survival rate/%	100	87.5	64.44
Number of molts	0	4	32
Molting rate/%	0	10	71.11
Initial body mass/g	9.54 ± 0.38	10.88 ± 0.37	11.19 ± 0.45
Final body mass/g	10.21 ± 0.47^a^	12.39 ± 0.43^b^	14.49 ± 0.56^c^
Average weight gain rate/%	1.79	3.57	10.29
Initial fatness/%	63.32 ± 0.67	61.35 ± 0.46	62.16 ± 0.62
Final fatness/%	63.62 ± 0.48^a^	62.50 ± 0.54^a^	59.67 ± 0.76^b^
Initial liver to body ratio/%	8.85	8.77	8.82
Final liver to body ratio/%	7.68 ± 0.31	7.87 ± 0.28	7.37 ± 0.34

**Note**: Values in the same row with different superscripted letters indicate significant difference (*p < 0.05*).

### 3.2 Intestinal microbiological analysis

#### 3.2.1 Results of ASV cluster analysis

**FIGURE 1:**
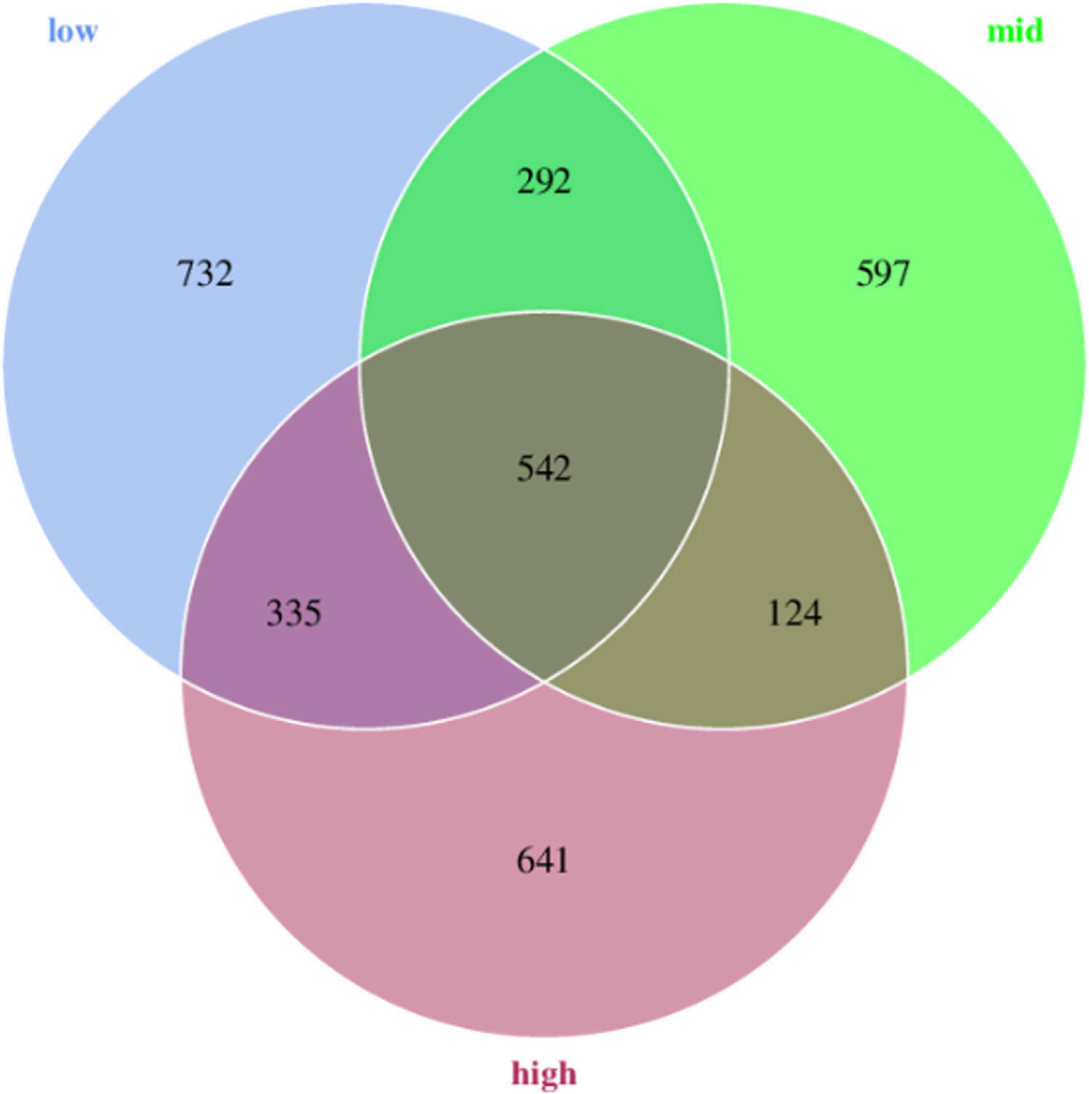
Venn diagram analysis of ASVs in the intestinal microorganisms of juvenile *E. sinensis* at different water temperatures.

A total of 3263 ASVs were obtained from 18 samples. Moreover, 1901, 1555, and 1642 representative ASVs were obtained in 15 °C, 23 °C and 30 °C water temperature groups, respectively. The results showed that with the increase in temperature, the number of ASVs initially decreased and gradually increased. The total number of ASVs in the three groups was 542, while the number of endemic ASVs was 732, 597, and 641 for the respective groups (Figure 1).

#### 3.2.2 Results of diversity analysis

**FIGURE 2:**
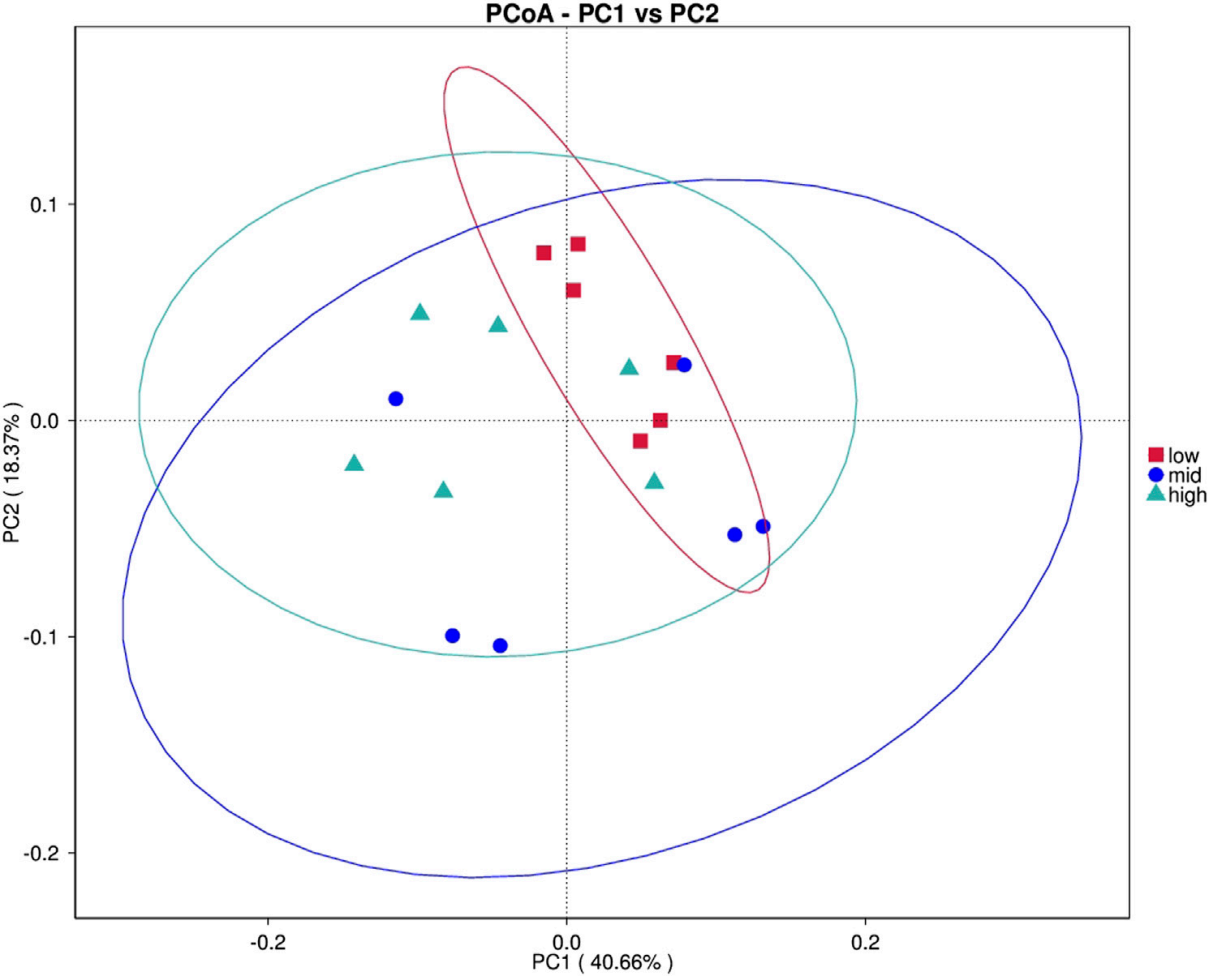
Principal coordinate analysis (PCoA).

After feeding for 30 days at different water temperatures, the PCoA of intestinal microbial composition of juvenile *E. sinensis* showed that the contribution of principal components to sample differences was 40.66% and 18.37% (Figure 2). Alpha diversity analysis showed that Simpson and Pielou_e indexes in the 23 °C water temperature group were significantly lower than those in the 15 °C and 30 °C groups (*p* < 0.05), while Simpson and Pielou_e indexes initially decreased and gradually increased, indicating that species diversity and evenness increased at first and then decreased with the increase in water temperature. There was no significant difference in the Chao and Shannon index among the three groups, however, all showed an increasing trend and decreased subsequently (Table 2).

**TABLE 2:** Alpha diversity of intestinal bacterial community of juvenile *E. sinensis* at different water temperatures.

Sample name	Low	Mid	High
Chao	597.1155 ± 65.58737	460.8863 ± 69.12549	506.5910 ± 58.05199
Dominance	0.0862 ± 0.0146^b^	0.1527 ± 0.0233^a^	0.0713 ± 0.01165^b^
Goods_coverage	0.9995 ± 0.00022	0.9995 ± 0.00022	0.9998 ± 0.00017
Observed_otus	590.5000 ± 66.19806	453.3333 ± 71.55495	502.8333 ± 57.33958
Pielou_e	0.5865 ± 0.0145^a^	0.5068 ± 0.02461^b^	0.5938 ± 0.0239^a^
Simpson	0.9138 ± 0.0146^a^	0.8473 ± 0.0233^b^	0.9287 ± 0.01165^a^
Shannon	5.3807 ± 0.2155	4.4405 ± 0.31849	5.3128 ± 0.2925

**Notes**: Values in the same row with different superscripted letters indicate significant difference (*p* < 0.05).

#### 3.2.3 Composition of intestinal microflora of juvenile *E. sinensis* under different water temperatures

**FIGURE 3:**
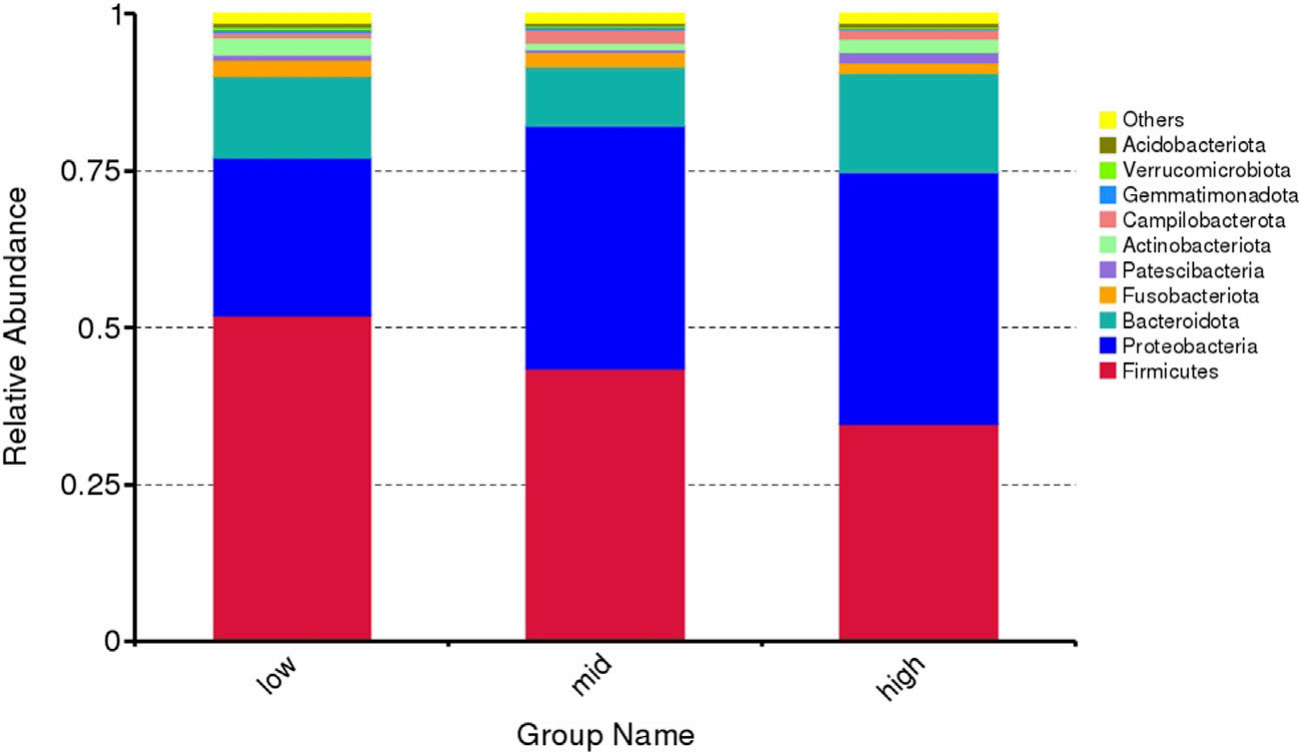
Microflora abundance at the phylum level of intestinal microorganisms in juvenile *E. sinensis* at different water temperatures.

The top 10 microorganisms at the phylum level in the intestinal abundance of juvenile *E. sinensis* reared at different water temperatures include Firmicutes, Proteobacteria, Bacteroidota, Fusobacteriota, Patescibacteria, Actinobacteriota, Campylobacterota, Gemmatimonadota, Verrucomicrobiota, and Acidobacteriota. The dominant flora of the three groups was Firmicutes, Proteobacteria, and Bacteroidota, which accounted for 90.75% of the intestinal flora of juvenile *E. sinensis* (Figure 3). There was no significant difference in the composition and abundance of intestinal microflora among different water temperature groups; however, the abundance of Firmicutes decreased with the increase in water temperature, while the abundance of Proteobacteria changed in direct proportion to the water temperature. The abundance of Bacteroidota decreased at first and gradually increased with the increase in water temperature.

**FIGURE 4:**
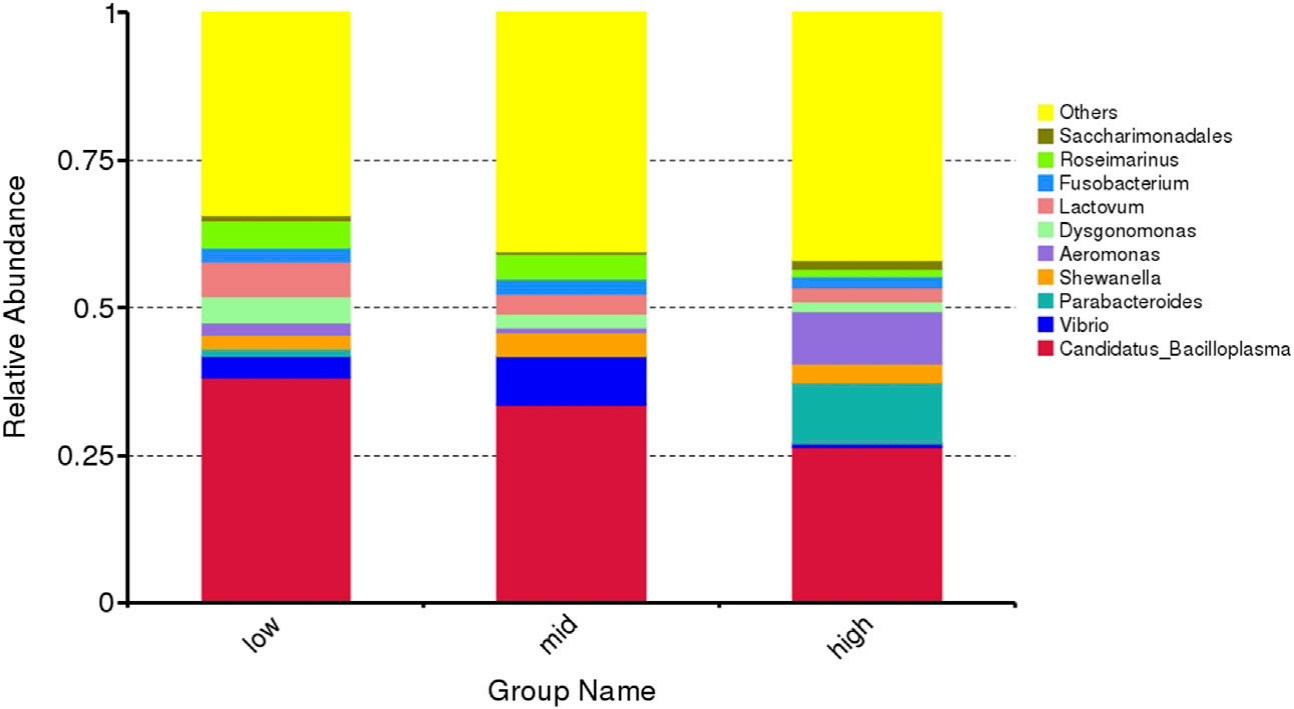
Microflora abundance at the genus level of intestinal microorganisms in juvenile *E. sinensis* at different water temperatures.

**FIGURE 5:**
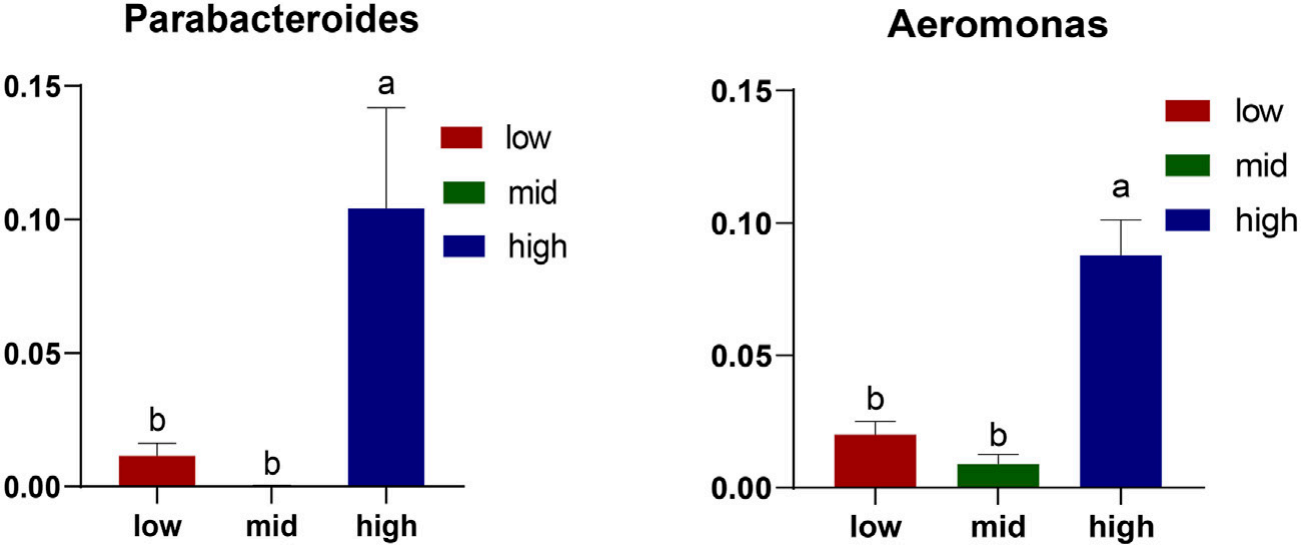
Differential microorganisms at the genus level. The results are expressed as mean ± SEM (*n* = 6); different letters indicate significate differences among different groups (*p < 0.05*).

The top 10 abundant genera are *Candidatus Bacilloplasma*, *Vibrio*, *Parabacteroides*, *Shewanella*, *Aeromonas*, *Dysgonomonas*, *Lactovum*, *Fusobacterium*, *Rosmarinus*, and *Saccharimonadales* (Figure 4). Among them, the abundance of *Parabacteroides* and *Aeromonas* in crab intestine in the 30 °C water temperature group was significantly higher than that in the 15 °C and 23 °C groups (Figure 5, *p* < 0.05).

#### 3.2.4 Analysis of the difference of the intestinal microbial community

**FIGURE 6:**
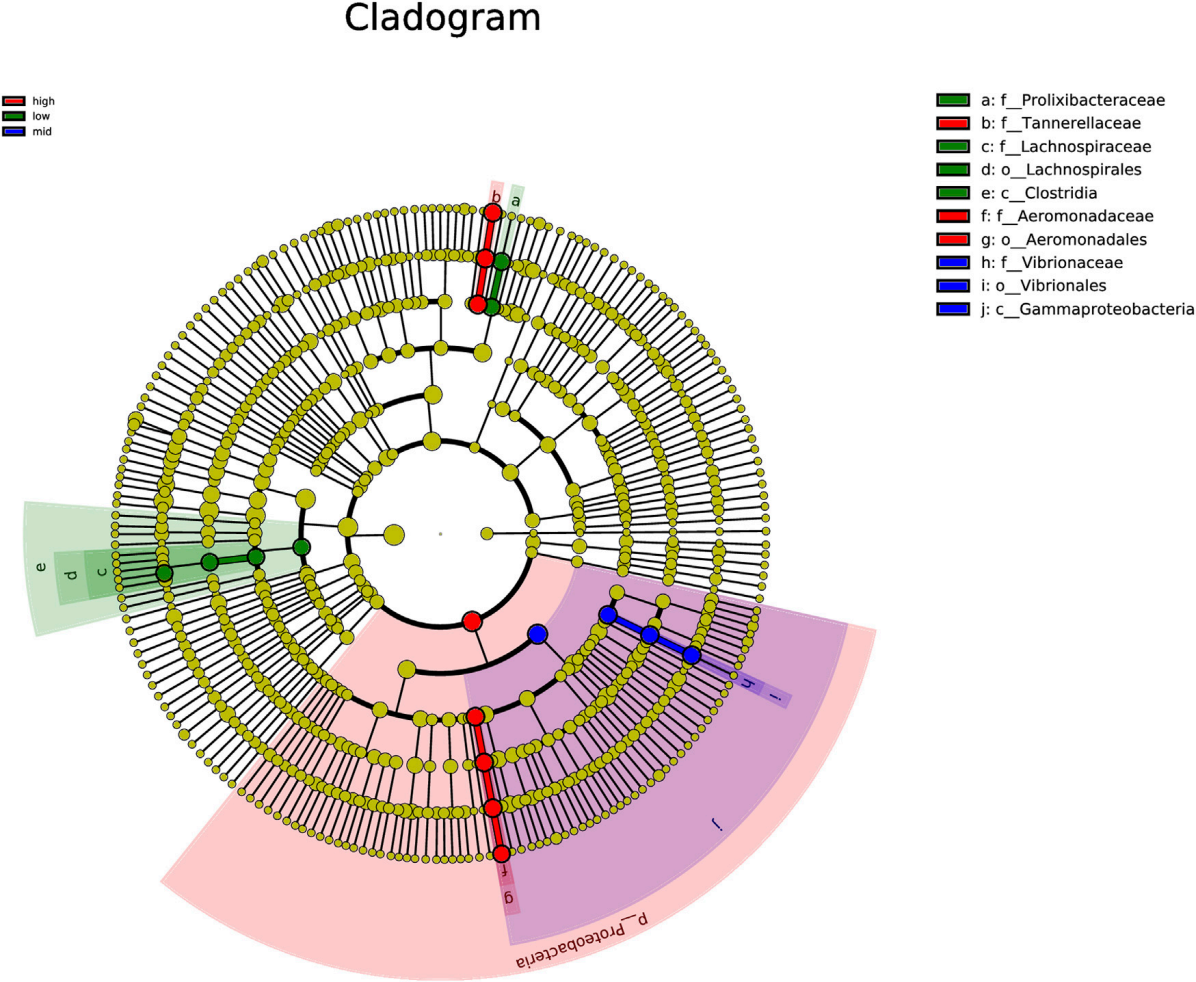
Clade map of gut microbiota.

Using LEfSe algorithm analysis, the evolutionary branch diagram of microorganisms in the intestine was obtained (Figure 6). The results showed that the 30 °C water temperature group (high) was the most differentially abundant, followed by 15 °C (low), with the 23 °C (mid) water temperature group having the least abundant.

**FIGURE 7:**
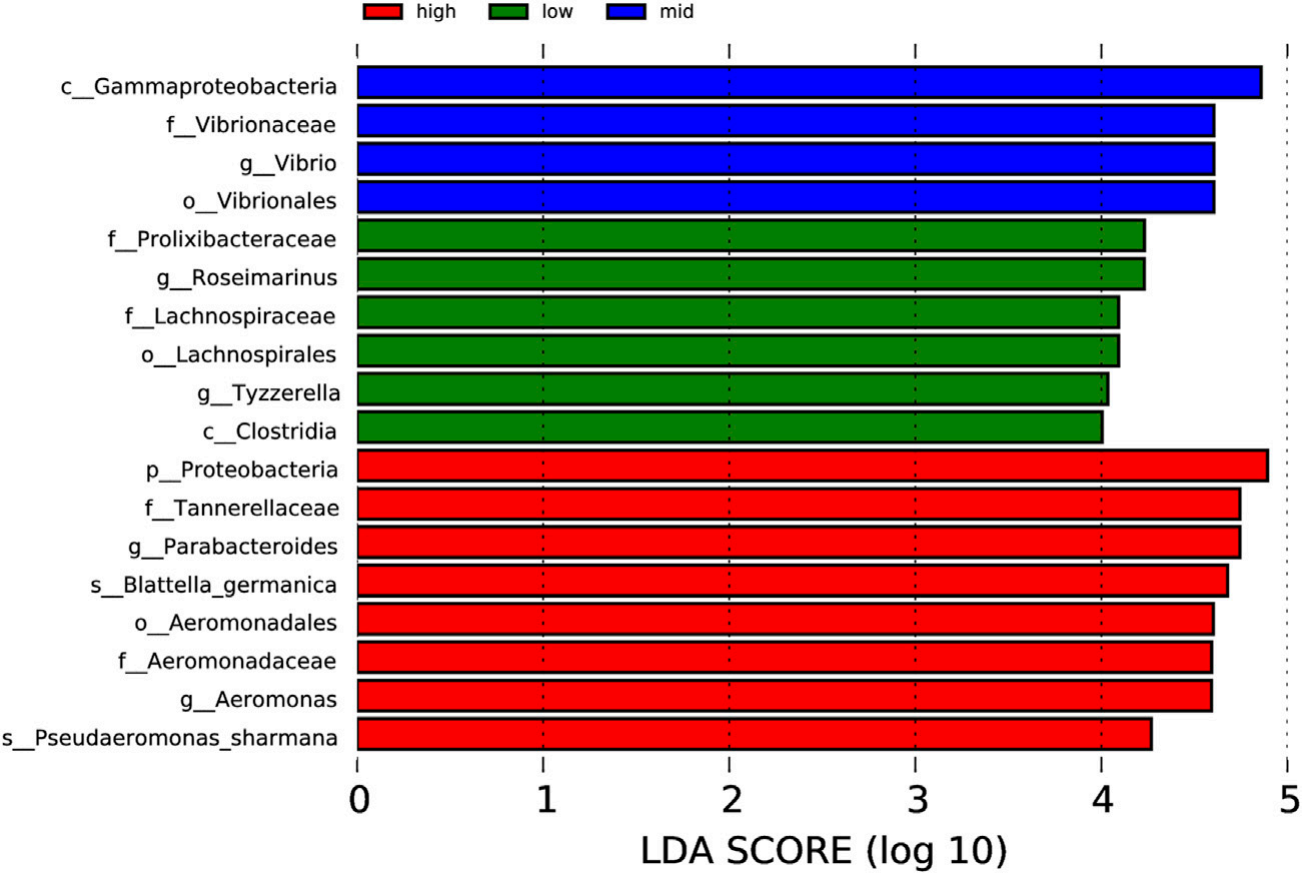
Column chart of LDA value distribution.

Through the analysis of microorganisms with an LDA value <4 in each group (Figure 7), the 30 °C water temperature group (high) is the group with the most number of microbial species (eight species), which include Proteobacteria, Tannerellaceae, *Parabacteroides*, *Blattella germanica*, Aeromonadales, Aeromonadaceae, *Aeromonas*, and *Pseudaeromonas sharmana.* This is followed by the 15 °C water temperature group (low), with six species namely Prolixibacteraceae, *Rosmarinus*, Lachnospiraceae, Lachnospiraceae, *Tyzzerella*, and Clostridia. In the 23 °C water temperature group (mid), there were four species, namely, Gammaproteobacteria, Vibrionaceae, *Vibrio*, and Vibrionales.

#### 3.2.5 Prediction of the function of intestinal microflora

**FIGURE 8:**
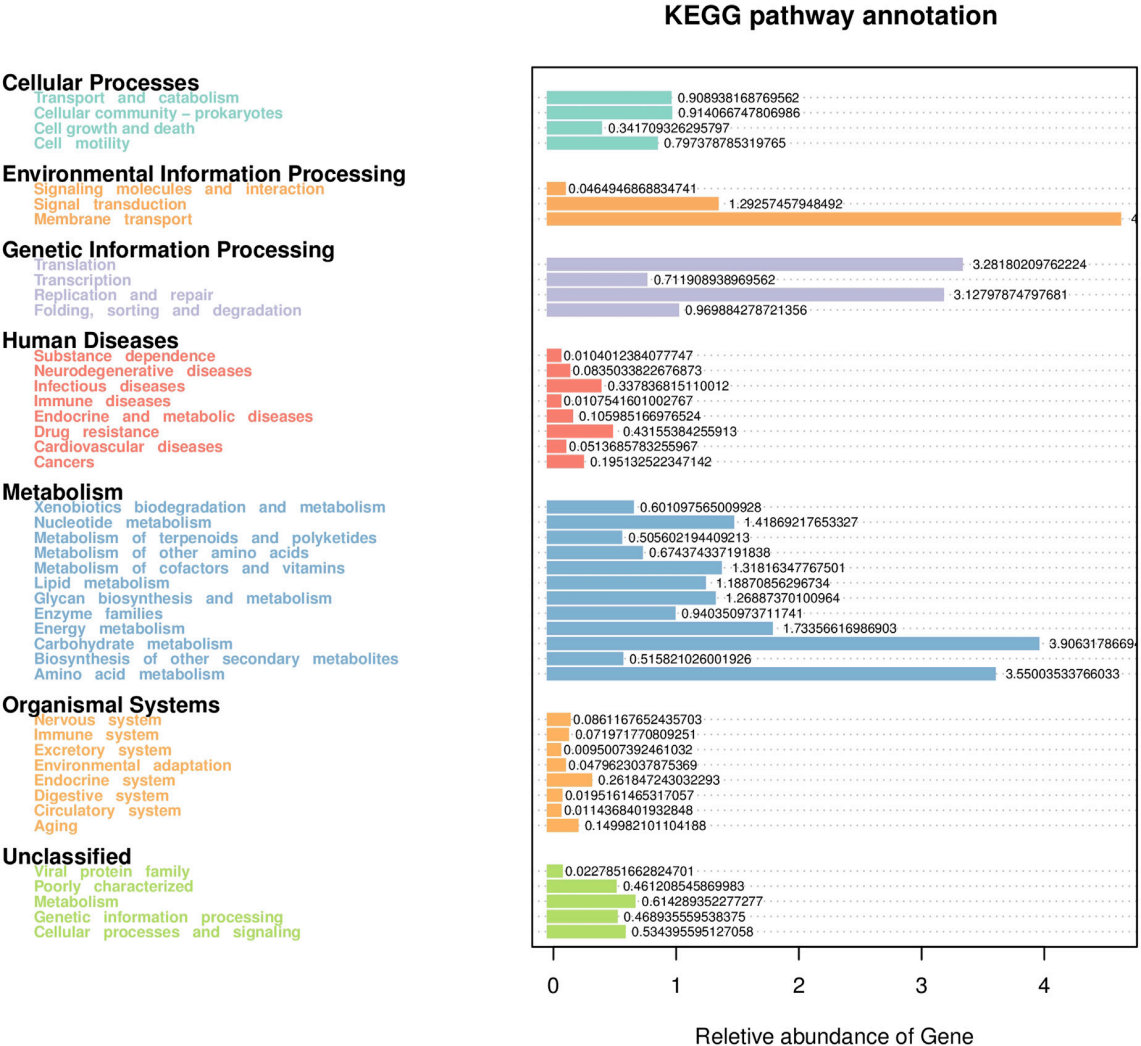
Statistical map of gene prediction results reveals the annotated proportion of genes.

**FIGURE 9:**
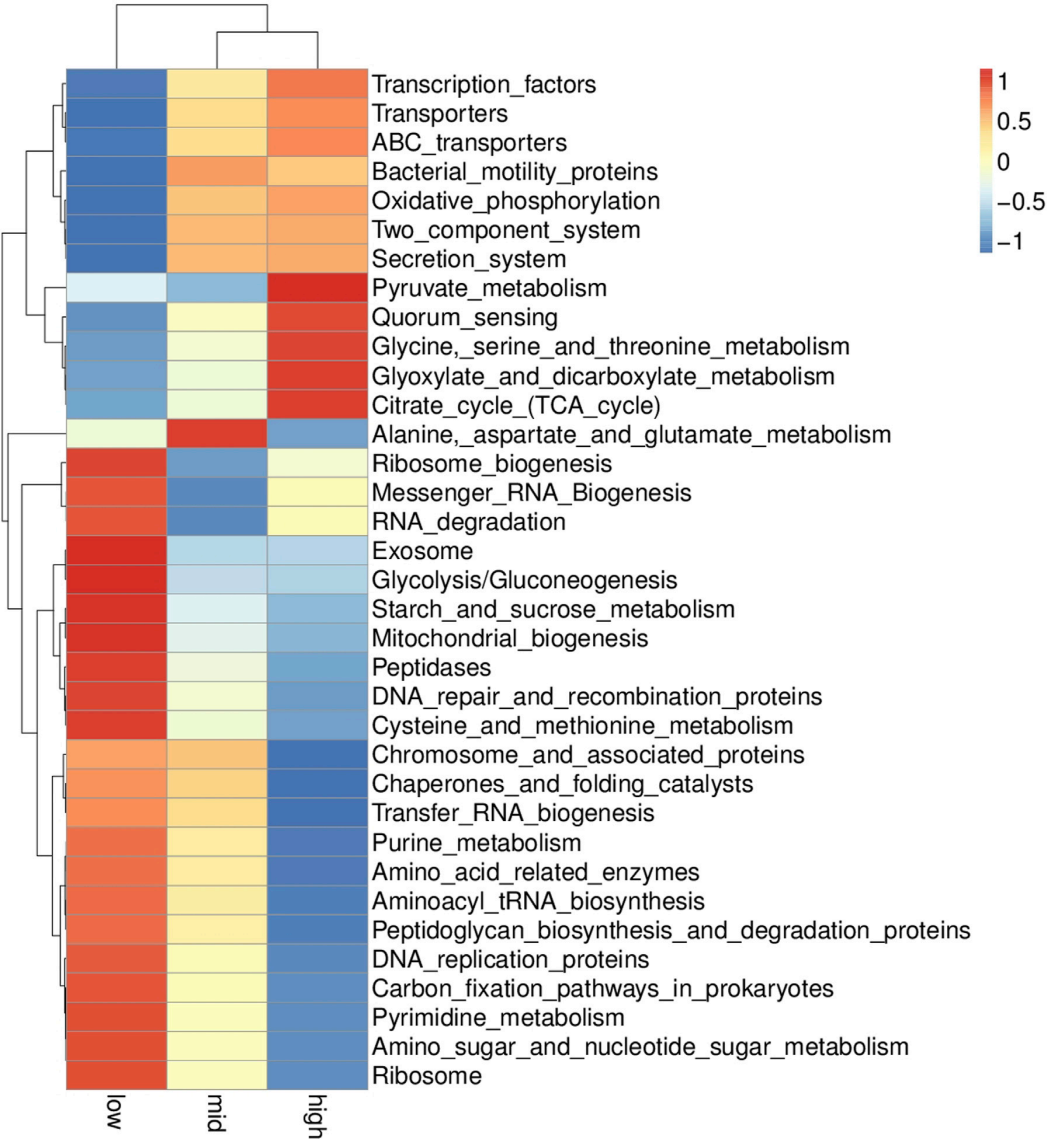
KEGG level 3 functional relative abundance clustering heatmap.

**FIGURE 10:**
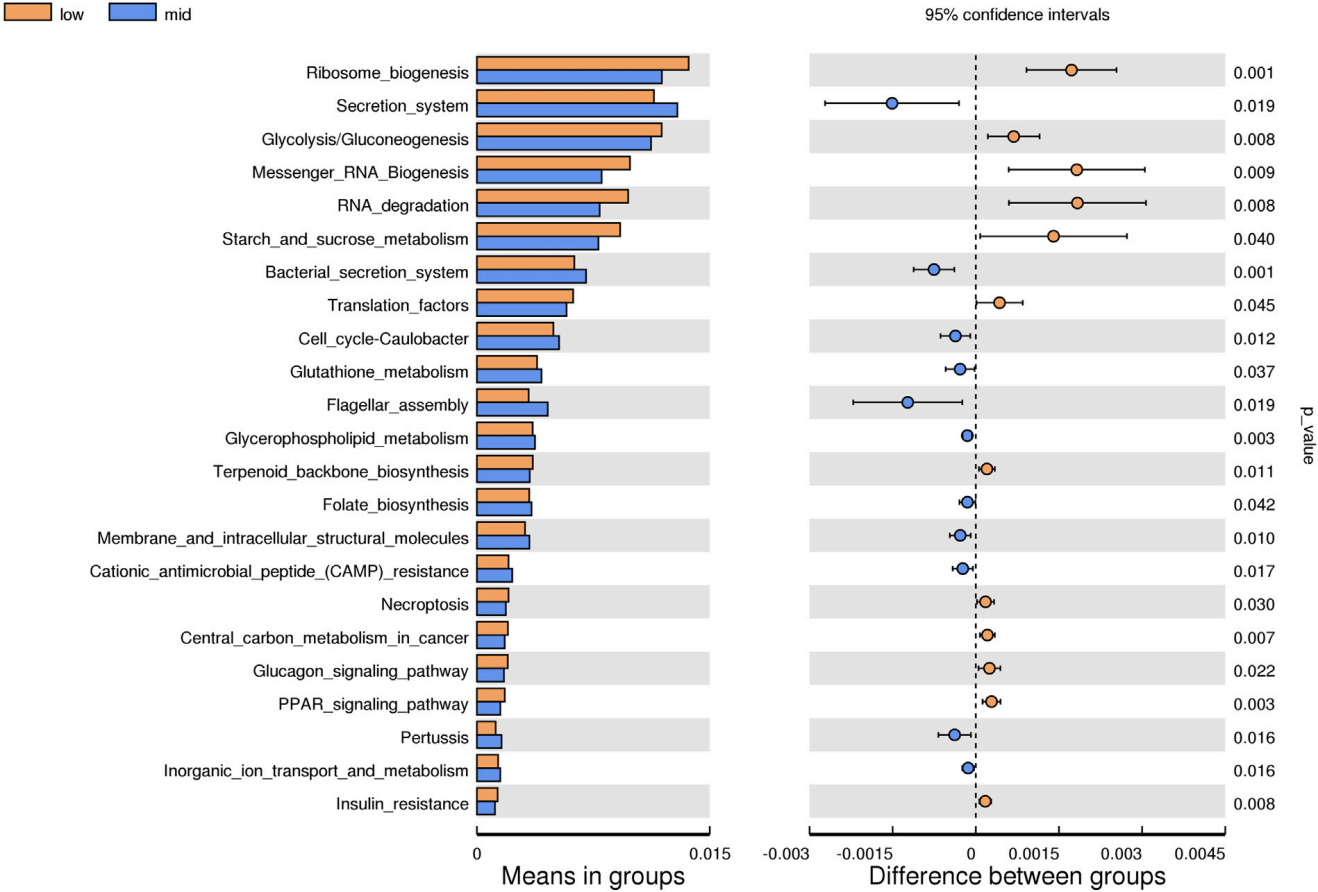
KEGG level analysis of predicted functional differences between low group and mid group.

**FIGURE 11:**
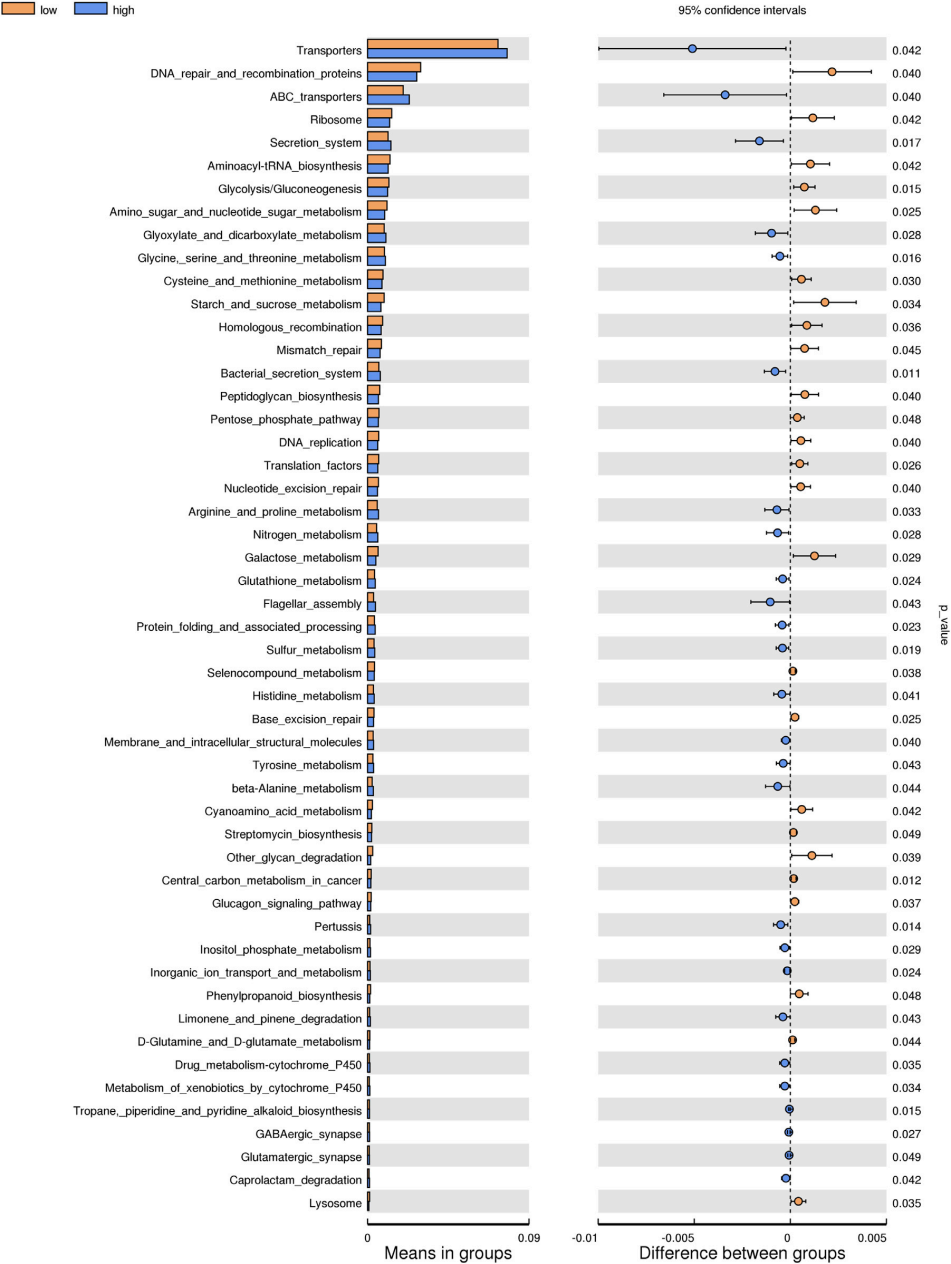
KEGG level analysis of predicted functional differences between low group and high group.

**FIGURE 12:**
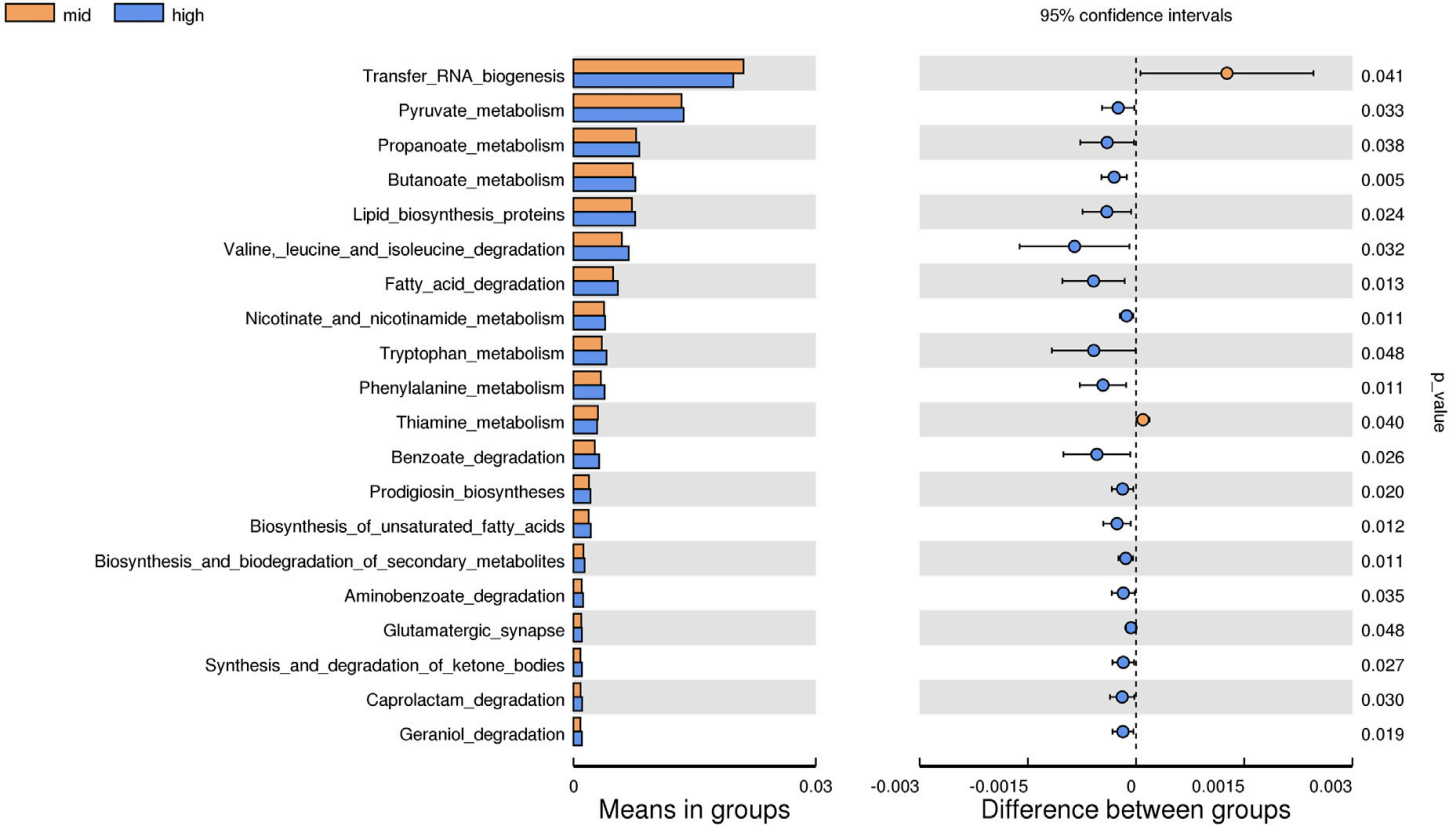
KEGG level analysis of predicted functional differences between mid group and high group.

Based on the KEGG database, the potential function of intestinal flora of juvenile *E. sinensis* in different water temperature groups was predicted by Tax4Fun. The results showed that there was no significant difference in the prediction of main functions among the three water temperature groups. In terms of “Metabolism,” intestinal microorganisms of juvenile *E. sinensis* play an important role in “Carbohydrate metabolism” and “Amino acid metabolism.” The main roles in “Genetic Information Processing” are “Translation” and “Replication and repair;” “Environmental Information Processing” mainly involves “Membrane transport” and “Signal transduction.” However, a minimal role involving “Signaling molecules and interaction” was detected (Figure 8). At KEGG level 3, the difference of gene function was shown by a heatmap (Figure 9). Function prediction revealed that the intestinal microflora in the 15 °C water temperature group was higher in “Exosome,” “Glycolysis/Gluconeogenesis,” and “Mitochondrial biogenesis” pathways. Moreover, the “Alanine, aspartate, and glutamate metabolism” pathway was more enriched in the 23 °C water temperature group. Meanwhile, the intestinal microflora in the 30 °C water temperature group was highly enriched in “Pyruvate metabolism,” “Glyoxylate and dicarboxylate metabolism,” and “Citrate cycle (TCA cycle)” pathways. A *t*-test analysis was performed on the functional differences of KEGG level 3 among the three water temperature groups (Figures 10–12). The results indicated that 23 pathways mainly including “Ribosome_biogenesis”, “Secretion_system”, and “Glycolysis/Gluconeogenesis” showed a maximum difference between the 15 °C and the 23 °C water temperature groups (*p* < 0.05). In total, 50 pathways mainly including “Transporters,” “DNA_repair_and_recombination_proteins,” and “ABC_transporters” showed a maximum difference between the 15 °C and the 30 °C water temperature groups (*p* < 0.05). Furthermore, 20 pathways mainly including “Transfer_RNA_biogenesis,” “Pyruvate_metabolism,” and “Propanoate_metabolism” showed a maximum difference between the 23 °C and 30 °C water temperature groups (*p* < 0.05).

## 4 Discussion

Temperature is one of the most important external factors affecting the growth and development of crustaceans, as well as the culture of juvenile *E. sinensis*. In this study, the effects of three different water temperatures on the growth and intestinal microorganisms of juvenile *E. sinensis* were studied. The results showed that the molting and weight gain rate of experimental crabs increased significantly with the increase in water temperature, which was consistent with the results of Yuan et al. (2017), and reflected the promoting effect of temperature on the growth and development of crustaceans. However, with the increase in temperature, the survival rate of experimental crabs decreased substantially. A higher water temperature can change the concentration of ammonia nitrogen and total nitrogen, which can spoil the aquaculture water body, leading to low survival and mortality among *E. sinensis* (Peng et al., 2019). *E. sinensis* may become weaker and be at a higher risk of mortality if the water temperature exceeds the physiological bearing limit of *E. sinensis*. Studies have shown that in a certain temperature range, the higher the temperature, the more exuberant the metabolism and gonadal development of *E. sinensis* will be, leading to precocious puberty, thus inhibiting its growth (Li et al., 2011). With the increase in temperature, the fatness and liver-body ratio of experimental crabs showed a downward trend, which confirmed that higher temperature can lead to the occurrence of precocious puberty, thus affecting the size of adult crabs. Since this experiment was performed under laboratory conditions, the water temperature is constant, which better reflects the effect of temperature on the growth of the juvenile *E. sinensis*. Based on our findings, the optimum water temperature for the growth of juvenile *E. sinensis* is approximately between 23 °C and 30 °C.

Intestinal microorganisms form a colony in the intestinal tract and play an important role in the growth and development, physiological balance, intestinal development, metabolism, and immune regulation of the host (Kwong and Moran, 2016; Zhang et al., 2019; Yukgehnaish et al., 2020). Temperature has an important effect on the composition and metabolites of intestinal microorganisms. It was found that both low- and high-temperature heat stress could change the abundance of host intestinal microflora (Liu et al., 2020). Ramos-Romero et al. (2020) found that low temperature increased the abundance of the *Clostridium subgroup* and *Enterobacter*, and decreased the abundance of *Lactobacillus* in rats. Kohl and Yahn (2016) found that a warm environment significantly increased the relative abundance of Planctomycetes and *Mycobacterium* in tadpole intestinal microflora. In this study, the results of alpha diversity analysis showed that the Chao index values of the 23 °C and 30 °C water temperature groups were lower than that of the 15 °C group, which indicated that the abundance of intestinal microorganisms decreased with the increase in temperature. This suggests that lower temperature was more beneficial to the survival and reproduction of intestinal microorganisms, which was consistent with the results of Zhang et al. (2022). However, the Simpson and Pielou_e indexes of the 23 °C group were significantly lower than those of 15 °C and 30 °C water temperature groups. The diversity and evenness of intestinal microorganisms in juvenile *E. sinensis* decreased at first and then increased with the increase in water temperature, but the numerical difference was small. We speculated that compared with the ambient temperature of 15 °C, the intestinal microbial diversity and evenness of the 23 °C group decreased slightly due to temperature stress, while the 30 °C group was close to the optimum survival temperature of juvenile crabs. Therefore, the microbial diversity and evenness of the 30 °C group are higher than those of the 23 °C group, and the specific reasons need to be further studied. Concurrently, the growth of the juvenile *E. sinensis* was not optimal at 23 °C, indicating that a water temperature of 23 °C was not ideal for the growth of the juvenile *E. sinensis*. Optimal proportion of microflora but not higher abundance and diversity can be indicative of better growth for juvenile *E. sinensis* (Chen et al., 2021b), making intestinal microorganisms reach a stable state in the host, thus promoting its growth and development.

The abundance and proportion of Firmicutes and Bacteroidota flora, two dominant intestinal microflora, play an important role in the health and metabolism of the host (Singh et al., 2017; Lin et al., 2019). In mammals, energy accumulation is related to the distribution of intestinal flora. When intestinal Firmicutes is more abundant than Bacteroidota, the host can absorb and utilize the heat in food more effectively (Mazier et al., 2021). In this study, Firmicutes are the most abundant in the three temperature groups, followed by Proteobacteria and Bacteroidota. However, with the increase in temperature, the abundance of Firmicutes decreased, while Proteobacteria increased gradually. The abundance of Proteobacteria in the intestines of the crabs in the three respective water temperature groups was 25.1%, 38.56%, and 40.09%. Studies have shown that Proteobacteria is a dominant microbiota of the intestinal tract of the crustaceans (Zhang et al., 2014; Sun et al., 2018). As a group of Gram-negative bacteria, it plays an important role in nutritional circulation and mineralization of organic compounds (Kirchman, 2002; Liu et al., 2021). Concomitantly, Proteobacteria, including several pathogens, is the largest phylum of bacteria (Han et al., 2019), and thus, its continuous accumulation can represent the health of the host or the imbalance structure of intestinal microorganisms (Shin et al., 2015). Sun et al. (2018) speculated that the increase in relative abundance of Proteobacteria may be the reason for the poor growth performance of *Portunus trituberculatus*. We believe that in the early stage, with the increase in water temperature, the content of beneficial bacteria in the intestines of juvenile *E. sinensis* increases, promoting their digestion and absorption of food, and accelerating their growth. Concurrently, with the increase in stress time, a relatively higher temperature can lead to the rapid reproduction of pathogens, destroying the balance of intestinal microorganisms of the juvenile *E. sinensis.*

*Candidatus_Bacilloplasma* is the most abundant genus in the intestine of juvenile *E. sinensis*. Its abundance in the 15 °C and 23 °C water temperature groups is higher than that in the 30 °C water temperature group. Some studies have shown that the significant increase in *Candidatus_Bacilloplasma* and *Phascolarctobacterium* and the decrease in *Paracoccus* and *Lactococcus* may lead to white feces syndrome (WFS) of the Pacific white shrimp (Kostanjsek et al., 2007; Hou et al., 2018). Moreover, the abundance of *Vibrio* in the 15 °C and 23 °C groups was significantly higher than that in the 30 °C water temperature group. Some studies have shown that the presence of *Vibrio* may lead to intestinal diseases (Almagro-Moreno et al., 2015; Baker-Austin et al., 2018). The intestinal diseases caused by these pathogenic bacteria have a considerable impact on the growth and development of crabs, which is consistent with the growth of crabs in the 15 °C and 23 °C groups. The abundance of *Parabacteroides* and *Aeromonas* in crab intestine in the 30 °C water temperature group was significantly higher than that in the 15 °C and 23 °C groups (*p* < 0.05). *Parabacteroides* is a group of gram-negative anaerobes, which usually colonized the gastrointestinal tract of many species. Currently, the genus consists of 15 species. *Parabacteroides distasonis* is a typical strain of *Parabacteroides*, which plays an important role in alleviating host obesity, metabolic dysfunction (Wang et al., 2019), and enteritis (Kverka et al., 2011). This can promote the growth of crabs in the 30 °C water temperature group. *Aeromonas* is a facultative anaerobe with an optimal growth temperature of 30 °C. It can cause several intestinal diseases in humans and animals. Studies have shown that injecting *Aeromonas* into *Ictalurus punctatus* may lead to septicemia and can seriously affect the health of the host (Zhang et al., 2016; Ran et al., 2018). We speculate that this is an important reason for the higher mortality of crabs in the 30 °C water temperature group.

The results of predicting the gene function of intestinal microorganisms in juvenile *E. sinensis* showed that the functional diversity of intestinal microflora in the three water temperature groups was similar and mainly concentrated in metabolism, including carbohydrate and amino acid metabolism, which was consistent with other crustacean studies (Sun and Xu, 2021; Yan et al., 2021). Past studies have shown that Firmicutes, Proteobacteria, Actinobacteria, and *Bacteroides* play important roles in carbohydrate fermentation, polysaccharide catabolism, and amino acid and protein utilization (Flint et al., 2012; Flint et al., 2015). This is consistent with the results that the dominant flora of the three water temperature groups is Firmicutes, Proteobacteria, and Bacteroidota. In this study, the pyruvate metabolism, glyoxylate and dicarboxylate metabolism, and citrate cycle were more abundant in the 30 °C water temperature group, which might provide more energy for growth and other physiological activities (Zhang et al., 2020). Although the main functional diversity of intestinal flora in the three water temperature groups was similar, there were still differences in the effects of water temperature on functional pathways such as metabolism, immunity, and growth among each group, either promoting or inhibiting. Thus, our data indicated that different water temperatures can affect the composition and function of intestinal microorganisms. In addition, information regarding the function of intestinal microbiota in crustacean is currently limited and further investigation needs to be carried out in future study.

## 5 Conclusion

In summary, the results of this study show that temperature has a substantial effect on the growth and development of juvenile *E. sinensis*. Crabs raised at 15 °C grow slowly and can further grow with the increase in temperature. However, when the temperature reaches 30 °C, a substantial number of crabs may face mortality. Concomitantly, different temperatures alter the intestinal flora composition and function of juvenile *E. sinensis*, and high temperature can lead to the proliferation of pathogenic bacteria. Therefore, water temperature between 23 °C and 30 °C is most beneficial to the growth of juvenile *E. sinensis*.

## Data Availability

The original contributions presented in the study are included in the article/supplementary material; further inquiries can be directed to the corresponding author.
